# Mitochondrial Glucocorticoid Receptors and Their Actions

**DOI:** 10.3390/ijms22116054

**Published:** 2021-06-03

**Authors:** Ioanna Kokkinopoulou, Paraskevi Moutsatsou

**Affiliations:** Department of Clinical Biochemistry, Medical School, University General Hospital “ATTIKON”, National and Kapodistrian University of Athens, 124 62 Athens, Greece; iwanna-k@med.uoa.gr

**Keywords:** glucocorticoid receptor, mitochondria, mitochondrial glucocorticoid receptor, glucocorticoids, apoptosis, stress

## Abstract

Mitochondria are membrane organelles present in almost all eukaryotic cells. In addition to their well-known role in energy production, mitochondria regulate central cellular processes, including calcium homeostasis, Reactive Oxygen Species (ROS) generation, cell death, thermogenesis, and biosynthesis of lipids, nucleic acids, and steroid hormones. Glucocorticoids (GCs) regulate the mitochondrially encoded oxidative phosphorylation gene expression and mitochondrial energy metabolism. The identification of Glucocorticoid Response Elements (GREs) in mitochondrial sequences and the detection of Glucocorticoid Receptor (GR) in mitochondria of different cell types gave support to hypothesis that mitochondrial GR directly regulates mitochondrial gene expression. Numerous studies have revealed changes in mitochondrial gene expression alongside with GR import/export in mitochondria, confirming the direct effects of GCs on mitochondrial genome. Further evidence has made clear that mitochondrial GR is involved in mitochondrial function and apoptosis-mediated processes, through interacting or altering the distribution of Bcl2 family members. Even though its exact translocation mechanisms remain unknown, data have shown that GR chaperones (Hsp70/90, Bag-1, FKBP51), the anti-apoptotic protein Bcl-2, the HDAC6- mediated deacetylation and the outer mitochondrial translocation complexes (Tom complexes) co-ordinate GR mitochondrial trafficking. A role of mitochondrial GR in stress and depression as well as in lung and hepatic inflammation has also been demonstrated.

## 1. Mitochondrial Form and Function

Mitochondria are multifunctional life-sustaining organelles that contain several identical copies of their own genome—the mitochondrial DNA (mtDNA). They are present in almost every eukaryotic cell type, except red blood cells, and they exhibit several features of their bacterial origin [1]. They are surrounded by inner and outer mitochondrial membranes, which enclose the intermembrane space and the mitochondrial matrix [2]. Mitochondria have long been considered as the ‘energy powerhouse of the cell’, owing to their ability to produce energy in the form of ATP, which is carried out by mitochondrial oxidative phosphorylation system (OXPHOS) through oxidation of sugars, fats, and proteins. Beyond ATP production, mitochondria participate in the biosynthesis of amino acids, lipids, hemes, purines, and steroid hormones, control intracellular Ca^2+^ metabolism and signaling, generate reactive oxygen species (ROS), and regulate thermogenesis and programmed cell death. [3,4,5]. Moreover, mitochondria are considered the key components of the stress response owing to their role in energy production as well as their capacity to generate signals that promote the adaptive response to stressors [5,6].

**Figure 1:**
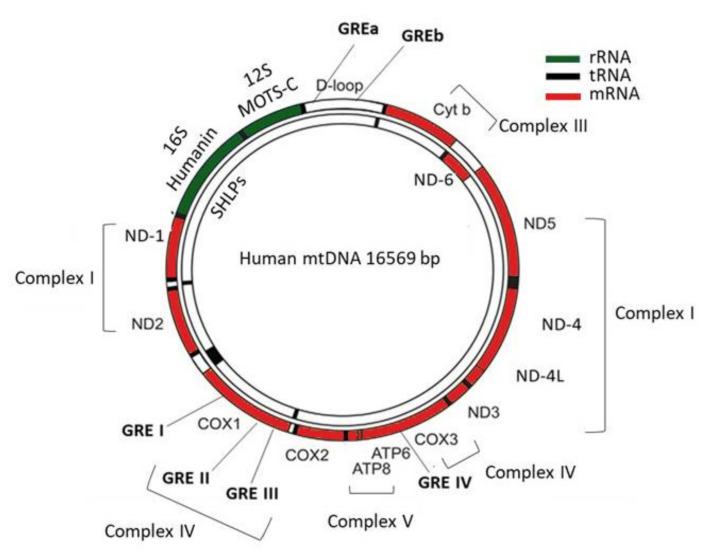
The human mitochondrial genome. The human mitochondrial genome consists of a circular, double-stranded DNA of 16.569 bp. It encodes two ribosomal RNAs (12S and 16S), 22 tRNAs, and 13 mitochondrial proteins involved in the respiratory complexes; *ND1, ND2, ND3, ND4, ND4L, ND5, ND6* encode subunits of the complex I, *COX1, COX2, COX3* of the complex IV, *CYTB* of the complex III, and *ATP6, ATP8* of the complex V. Three mitochondrial GREs reside in *COX1* gene, one in *COX3* and two within the D-loop. MOTS-c is encoded by ORFs within 12S rRNA while Humanin and SHLPs by ORFs within 16S rRNA.

In humans, the maternally inherited mtDNA consists of a circular, intronless, double-stranded DNA of about 16.6 kb. Most of the mitochondrial proteins are encoded by the nuclear genome, except for the 13 mitochondrially encoded genes, namely *NADH ubiquinone oxidoreductase core subunit 1–6 (ND-1, ND-2, ND-3, ND-4, ND-4L, ND-5, ND-6), Cytochrome b (CYT-B), Cytochrome c oxidase 1–3 (COX-1, COX-2, COX-3),* and *ATP synthase membrane subunit 6 and 8 (ATP-6, ATP-8)*, which provide the essential protein subunits of the respiratory complexes I, III, IV and V, 22 mitochondrial tRNAs, and 2 ribosomal RNAs (12S and 16S) [7]. In addition, a noncoding region of the mtDNA, the D-loop, is essential for both mtDNA replication and transcription (Figure 1) [8].

The coordination between nuclear and mitochondrial genes is required for the controlled mitochondrial biogenesis and function. Mutations in either mitochondrial or nuclear genes that encode the mitochondrial proteins can affect mitochondrial metabolism and they have been linked to the development of numerous neurodegenerative and metabolic disorders [9]. Recent studies have focused their interest on mitochondria-derived peptides (MDPs), which consist of a new class of peptides, encoded by short open reading frames (ORFs) within mitochondrial genes, including MOTS-c (mitochondrial open reading frame of the 12S rRNA-c peptide), humanin and small humanin-like peptide 1-6 (SHLP1-6) (Figure 1) [10,11,12]. Even though the exact role of the ORF-derived peptides remains unknown, studies have shown that these peptides participate in the regulation of mitochondrial function while their dysregulation has been associated with the development of metabolic and cardiovascular disorders [13,14].

## 2. Effects of Glucocorticoids on Mitochondria

Glucocorticoids (GCs) control a wide variety of physiological processes which require increased energy expenditure, and thus regulation of the mitochondrial energy metabolism is one of their major functions [5]. However, GC-induced mitochondrial effects are dependent on the treatment dose and exposure duration. Studies have revealed that acute and limited exposure cell stimulation with GCs augment the capacity of mitochondria to generate energy, through activation of the pre-existing respiratory chain components, enhancement of mitochondrial and nuclear gene expression, increase of mitochondrial membrane potential, prevention of programmed cell death and in high energy demands, increasing mitochondrial biogenesis as well as mitochondrial DNA content [15,16,17,18,19,20].GCs also improve the mitochondrial respiration in Duchenne Muscular Dystrophy mouse model through inducing calcium accumulation in skeletal muscle mitochondria [21].

The modulation of mitochondrial function by GCs is mediated by a biphasic manner; whereas short-term exposure to GCs serves a protective mechanism which is associated with induction of mitochondrial biogenesis and increase of enzymatic activity of the respiratory chain, long-term exposure to GCs causes respiratory chain dysfunction, decreased ATP production, increased ROS generation, mitochondrial structural abnormalities, abnormal mitochondria biogenesis, decreased mitochondrial membrane potential, increased sensitivity to cell death and telomere attrition [5,22,23]. GCs have been shown to reduce respiration control ratio through direct inhibition of cytochrome c oxidase activity in isolated rat kidney mitochondria as well as through inhibition of complex I and V activity in rat brain mitochondria [24,25]. GCs also inhibit calcium influx in C2C12 myocytes [26]. GCs exhibit biphasic effects on cortical neurons; short-term treatment with high or low doses and long-term treatment with low doses enhance mitochondrial oxidation, mitochondrial potential, and calcium holding capacity, while long-term treatment with high doses reverses the GCs-induced effects [27].

The first hypothesis that mitochondrial genome could be an additional site of GCs action had been pointed out in early studies by the Sekeris group, who identified that human and mouse mitochondrial sequences show partial homology to glucocorticoid response elements (GREs) [28]. Since then, studies have identified the presence of glucocorticoid receptor (GR) in mitochondria of different tissues and cell types, and they have confirmed the direct action of GCs on mitochondrial gene transcription through interaction of GR with mitochondrial GREs in parallel with their nuclear action [20,29].

The purpose of this review is to gain insight into the existing studies relevant to the area of mitochondrial GR and to provide current evidence regarding its role in the transcription of mitochondrially encoded OXPHOS genes as well as in mitochondrial function. Furthermore, this review deals with the up-to-date molecular mechanisms that are required for GR mitochondrial translocation as well as with the involvement of mitochondrial GR in apoptotic signaling pathways and disease development.

## 3. Mechanism of Action of Glucocorticoids–Glucocorticoid Receptor

The effects of GCs in the regulation of nuclear gene transcription are well known. GC availability is sustained by tissue-specific metabolic enzymes, namely the 11β-hydroxysteroid dehydrogenases (11β-HSDs). In particular, 11β-HSD1 catalyzes the conversion of inactive cortisone to active cortisol while 11β-HSD2 inactivates GCs through converting cortisol to cortisone [30]. At the cellular level, the action of GCs, with cortisol being the predominant endogenous GC, is mediated mainly by an intracellular protein called the human glucocorticoid receptor (hGR), which is ubiquitously expressed and belongs to the nuclear receptor superfamily of ligand-dependent transcription factors. It is encoded by *NR3C1* gene, and it is composed of three major functional domains; the N-terminal transactivation domain (NTD), the central DNA-binding domain (DBD), and the C-terminal ligand-binding domain (LBD) [31]. Alternative splicing and alternative initiation sites of GR mRNA are responsible for the generation of numerous receptor isoforms, including GRα, GRβ GRγ, GRA, and GRP.

The hGRα represents the classic GR isoform, while hGRβ acts as an inhibitor upon the transcriptional activity of hGRα [32]. Additionally, these isoforms are subjected to several post-translational modifications, including methylation, acetylation, nitrosylation, sumoylation, ubiquitination and phosphorylation, affecting the protein’s stability, function, localization, and interaction with other proteins [33,34]. Mutations and polymorphisms in the hGR gene have been associated with-generalized or tissue specific- increased (hypersensitivity) or decreased (resistance) glucocorticoid sensitivity [35]. The majority of hGR mutations have been correlated with the Primary Generalized Glucocorticoid Resistance or ‘Chrousos syndrome’ which consists of a rare, monogenic disorder associated with insensitivity to GCs and impaired glucocorticoid signaling [36]. In addition, hGR polymorphisms, such as *N363S, BclI, ER22/23EK, TthIIII*, seem to affect tissue sensitivity to GCs and they have been linked to obesity, insulin resistance, coronary artery disease, high cholesterol, and triglyceride concentrations, etc. [31].

The genomic effects of GCs involve the action of GRα. In its unliganded state, GRα is located inside the cytoplasm complexed with chaperone proteins, namely Hsp70, Hsp90 (heat shock proteins 70/90), p23 and immunophilins (FKBP51 and FKBP52). Upon ligand binding, GRα undergoes conformational changes and translocates to the nucleus, where it interacts with coregulators and binds to GREs in the promoter region of target genes, regulating their expression negatively or positively [37]. Alternatively, hGRα can modulate gene expression independently of binding to GREs, by interacting with proinflammatory transcription factors, such as nuclear factor κB (NF-κB) and activator protein-1 (AP-1) [32]. GCs via the GR-GRE transactivation mechanism exert detrimental effects on the metabolism while via the GR-NFκB trans-repression mechanism, they mediate their beneficial anti-inflammatory effects [38]. However, reports show that GR-GREs-dependent transactivation is essential in the anti-inflammatory activities of GR [39].

In addition to the genomic actions, rapid non-genomic GC actions can also occur, mediated by non-specific interactions with the cell membrane, specific interactions with cytosolic GRs (cGR) or membrane bound GRs (mGR), which can indirectly affect gene transcription, triggering the activation of kinase signaling pathways such as phosphoinositide 3-kinase (PI3K), protein kinase B (AKT), and mitogen-activated protein kinases (MAPKs) [40]. Particularly, studies have shown that mGR can trigger the rapid events through interaction with other membrane receptors, and especially G protein-coupled receptors (GPCRs) or can directly activates downstream intracellular signaling pathways [41].

## 4. Glucocorticoid Receptor Localization in Mitochondria

Sekeris et al. were among the first to suggest the hypothesis that the mitochondria could be a primary site of action of steroid hormones by identifying that human and mouse mitochondrial sequences show partial homology to glucocorticoid and estrogen response elements [28]. A few years later, Demonacos et al. revealed the presence of GR in rat liver mitochondria as well as its import from the cytoplasm into the mitochondria upon administration of Dexamethasone (Dex), supporting the hypothesis that GR can interact directly with the mitochondrial genome [42]. Subsequent work revealed that purified GR from rat liver cytosol and GR containing in mitochondrial extracts from Dex-induced mice bind with high specificity to six potential mitochondrial GREs-four localized within the *COX1,* and *COX3* genes and two within the D-loop region (Figure 1) [43]. Further supportive data demonstrated that the putative mitochondrial GREs (GREI, GREII, and GREIII) confer dexamethasone inducibility to plasmids carrying the thymidine kinase promoter linked to the CAT (chloramphenicol acetyltransferase) reporter gene and transfected into LATK-cells, enhancing the hypothesis of a direct action of GCs on mitochondrial gene transcription [44,45]. The presence of GR in mitochondria in other cell types, such as human Hela and Hep-2 cells as well as in cytoplasmic and synaptosomal mitochondria of rat brain has also been demonstrated [46,47]. Interestingly, the coincidence of an additional signaling pathway-in parallel with GR signaling-in synaptic mitochondria, the Brain-derived neurotrophic factor (BDNF)-via its receptor TrkB-can regulate mitochondrial function and synaptic plasticity in the brain by altering the overall cellular GR-mediated gene expression signature [48].

## 5. Direct Effects of Glucocorticoid Receptor on Mitochondrial Gene Transcription

The presence of the mitochondrial sequences similar to nuclear GREs and the localization of GR in mitochondria suggested the possibility of a direct mechanism of GR in the induction of mitochondrially encoded genes, in parallel to its effect on nuclear OXPHOS genes. In this regard, Koufali et al. investigated GR localization as well as mitochondrial-cytosolic GR trafficking after treatment with Dex and its antagonist RU486 in rat C6 glioma cells [49]. Their study revealed that Dex treatment led to decrease in mitochondrial GR levels in parallel with its increase in nucleus. Importantly in this study, the Dex-induced GR export from mitochondria was associated with elevated expression of the COX-1 protein levels, suggesting that the mitochondrial GR has a direct effect on mitochondrial transcription and may act as a negative transcriptional factor in C6 glioma cells. Treatment of the cells with the GR selective antagonist-RU486-reversed the Dex-induced GR export from mitochondria and blocked the increase in COX-1 levels, pointing out that the effect of Dex on COX-1 levels occurs via a GR-mediated pathway [49]. On the other hand, Dex treatment of Salamander retina’s Müller glia cells led to mitochondrial GR translocation alongside with inhibition of the glutamate-induced increase of mitochondrial NADH, confirming a direct action of steroid hormones on mitochondrial metabolism [50].

A few years later, an increasing number of studies provide further evidence of a direct effect of mitochondrial GR on mitochondrial gene transcription. Psarra & Sekeris demonstrated in HepG2 hepatocarcinoma cells, GR-specific binding to the regulatory D-loop region of the mitochondrial genome and revealed that Dex induces the expression of the mitochondrial transcription factors A, B1, and B2 (*TFAM, TFB1M, TFB2M*), the mitochondrial *12 S* and *16S* RNA, and several mitochondrially encoded OXPHOS genes, including *ND 1–4, CYTB, ATP 6* and *ATP 8*, and *COX I* [51]. Applying α-amanitin, the specific inhibitor of DNA-dependent RNA polymerase II, the Dex-induced effect on the mitochondrial genes was continued, whereas the Dex effect on transcription of the nuclear-encoded transcription factors was suppressed. Moreover, HepG2 cells overexpressing mitochondrially localized GR showed increased RNA synthesis, COX I protein expression, and mitochondrial ATP production, verifying a nuclear independent, direct action of GR on the induction of mitochondrial transcription [51].

Recently, Hunter et al., based on animal studies, investigated the effect of acute and chronic stress exposure on mtRNA expression as well as the effect of corticosterone (Cort)-dose exposure on GR mitochondrial D-loop binding [52]. Their data demonstrated that Cort treatment induces a dose-dependent association of GR with the D-loop of the mitochondrial genome; treated rats with high Cort levels showed lower GR binding on D-loop compared to rats who treated with moderate Cort levels. Furthermore, GR exhibited biphasic effects in rat hippocampus after short- and long-term stress exposure; acute stress led to decreased mtRNA expression of *ND1, ND3, ND6* and *ATP6* genes, while chronic stress induced a significant elevated expression of *ND6* gene [52]. Taken together, the abovementioned studies provide evidence of mitochondrial GR localization in various cell types and tissues and verify the nuclear-independent effect of GCs on mitochondrial genome. Importantly, mitochondrial GR seems to be associated with-a dose-dependent-D-loop binding on the mitochondrial genome while its import into mitochondria is correlated with concomitant changes in mtRNA gene expression which are dependent on duration of stress exposure [53]. Table 1 summarizes the mitochondrial GR localization in parallel with alterations in the expression of the mitochondrially encoded genes.

Despite the increasing number of studies supporting the direct role of mitochondrial GR on the expression of mitochondrially encoded OXPHOS genes, mechanistic studies which elucidate the processes involved in mitochondrial GR trafficking are limited. It has been suggested that an inducible cytosolic Ser endoprotease is required for its mitochondrial transport through the activation of cryptic mitochondrial-targeting signals [54]. GR is characterized by the presence of a Ser protease-like processing site, near to the N-terminus of the protein, which is highly conserved among human, rat, and mouse. COS cells transfected with GR cDNA exhibit-except for the full-length protein-a 93 kDa processed product associated with the mitochondrial fraction. Both endoprotease subunits p90 and p40 contain Ser-protease processing activity domains and they are essential for GR mitochondrial translocation [54]. It is important to notice that other nuclear transcription factors and steroid receptors can also translocate to the mitochondria, including ER (Estrogen Receptor), CREB (cAMP response element-binding protein), NFΚB (nuclear factor kappa-light-chain-enhancer of activated B cells), AP-1 (activator protein 1) and p53 [55,56]. Among them, p53 shares similar processing patterns with GR and requires the same Ser endoprotease for its mitochondrial transport [54].

Little is known about the specific GR isoforms which exhibit mitochondrial localization and probably participate in mitochondrial function. Interestingly, Psarra et al. revealed that only GRα isoform appears to be mitochondrially localized in hepatocarcinoma HepG2 and osteosarcoma SaOS-2 cells whereas GRβ was confined solely to the nucleus [57]. Increasing evidence supports that the mitochondrial function is regulated by membrane GR, cytosolic GR, and other GR isoforms as well. Recently, Morgan et al. demonstrated that a specific highly conserved GR isoform-GRγ-with striking membrane association, seems to act as regulator of nuclear genes encoding mitochondrial proteins and interacts with mitochondrial proteins [58].Furthermore, GRγ expression induced the increase of mitochondrial mass, oxygen consumption and ATP production in the absence of added ligand, suggesting a key role of this isoform on mitochondrial function and energy expenditure [58]. 

Desquiret et al. investigated the GR-mediated pathways in the functioning of the respiratory chain after short- and long- term Dex treatment in HepG2 cells [59]. More specifically, short-term Dex treatment decreased the activity of complexes I, and II and increased the activity of the complex III of the respiratory chain, while the overall functioning of the respiratory chain was not affected. Importantly, their study showed that short-term Dex effects on the activity of the respiratory complexes I, II, and III involve the activation of a G protein-coupled membrane glucocorticoid receptor in a p38MAPK-dependent manner. Long-term Dex treatment, on the other hand, maintained the decrease of complex I and II activity but also increased complex IV activity and quantity in parallel with a decrease in oxidative phosphorylation efficiency. In the long-term Dex effects, the coordination between membrane GR and cytosolic GR glucocorticoid signaling has been implicated. Since, the rapid short Dex effects are associated with non-genomic actions while long term Dex effects with genomic actions, these data implicate that the effects of GCs on mitochondrial oxidative metabolism are complex, time exposure-dependent and involve multiple regulatory pathways [59].

## 6. Mitochondrial Glucocorticoid Receptor and Apoptosis

The role of mitochondrial GR in apoptosis has been investigated in various cell types, including lymphocytes, neutrophils, thymus, neural and pheochromocytoma cells as well as in brain tissue. The contribution of Bcl-2 (B-cell lymphoma-2), Bag-1 (Bcl-2-associated athanogene), Hsp70/90 (Heat Shock Proteins 70/90) and HDAC6 (Histone Deacetylase 6) in mitochondrial GR translocation has also been evaluated.

### 6.1. Blood and Thymus Cells

Accumulating evidence indicates a significant correlation between mitochondrial GR translocation and sensitivity of different cell types and tissues to GC-induced apoptosis. Sionov et al., using T-lymphoid cell lines varying in sensitivity toward GCs, investigated the role of mitochondrial GR in GC-induced apoptosis [60]. Their study revealed that Dex induces GR translocation to the mitochondria in GC-sensitive, but not in GC-resistant T-cell lines. A putative non-cleavable mitochondrial localization signal was also defined to amino acids 558–580 of the GR ligand-binding domain [60]. 

Later, Talabér et al. revealed that Dex-treated CD4+CD8+ double-positive thymocytes were characterized by reduced mitochondrial membrane potential in parallel with GR translocation into the mitochondria [61].To elucidate the mitochondrial apoptotic pathway involved in GC-induced thymocyte apoptosis, Prenek et al. investigated the interactions between GR and Bcl-2 family proteins in Dex-treated mouse thymocytes, including Bak, Bim, and Bcl-xL [62]. Upon high dose GC treatment, the GR translocates to the mitochondria and enhances its interaction especially with Bim, thus leading to Bax activation and triggering mainly the intrinsic pathway caspase-cascade [62]. On the other hand, Madsen-Bouterse et al., using Dex-treated bovine blood neutrophils, revealed increased mitochondrial membrane stability, reduced caspase-9 activity, and delayed apoptosis, alongside with increased expression of the antiapoptotic A1/Bfl-1 (Bcl-2-related protein A1) and decreased expression of the pro-apoptotic Bak (Bcl-2 antagonist/killer 1) [63]. Treatment with RU486, the GR antagonism, diminished the Dex-induced effects, implicating GR activation in GC-mediated delayed apoptosis [63]. Taken together, the above studies support that mitochondrial GR has a crucial role in apoptosis-mediated pathways in blood cells and thymocytes.

### 6.2. Neural Cells and Brain Tissue

A great deal of evidence investigated the contribution of time and dose-exposure of GCs and mitochondrial transclocation of GR on mitochondrial function and neuronal viability. In this sense, Du et al. analyzed the dose and time effects of GCs on neuronal mitochondrial function such as mitochondrial oxidation, membrane potential, and mitochondrial calcium holding capacity known to be associated with the apoptotic process [27]. They showed that short-term treatment with low or high doses of Cort improved the mitochondrial function of cortical neurons whereas longer-term treatment with high-dose Cort attenuated mitochondrial function, suggesting that Cort regulates neuronal mitochondrial function in a biphasic manner (dose- and time-dependent). In the same study, Du et al. investigated the effects of GCs on kainic acid (KA)-induced neuronal cell apoptosis and they showed that treatment with low-dose Cort had a neuroprotective effect, whereas long-term and high-dose concentrations of Cort enhanced neurotoxicity, also revealing the biphasic effects of GCs on neuronal viability and apoptosis [27]. In order to elucidate the mechanisms underlying the biphasic effects of GCs on mitochondrial function and neuronal viability/apoptosis, they investigated time and dose effects of Cort on mitochondrial translocation of glucocorticoid receptors (GRs); their study revealed that both low and high doses of Cort enhanced the mitochondrial localization of GRs whereas after long-term treatment with high doses of Cort, mitochondrial localization of GRs is reduced, suggesting that GRs translocate into mitochondria in a dose- and time-dependent manner.

**Figure 2:**
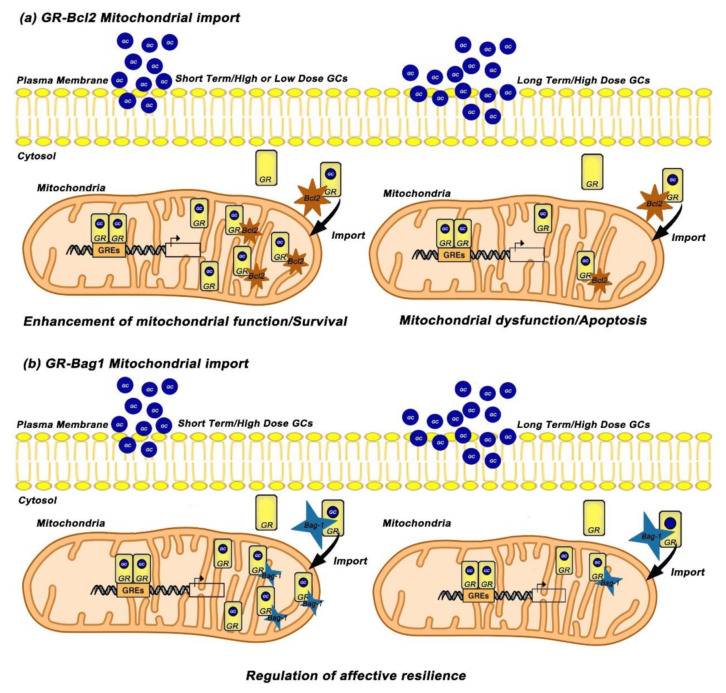
GCs exert biphasic effects on mitochondrial GR translocation and on the formation of GR/Bcl2 and GR/Bag-1 complexes in mitochondria of cortical neurons. (**a**) Short-term treatment with high or low doses of Cort induces the mitochondrial localization of GRs and leads to increase of the GR/Bcl-2 complex into mitochondria, thus enhancing mitochondrial function and neuronal viability. Long term treatment with high doses of Cort reduces the mitochondrial localization of GRs and downregulates GR/Bcl-2 complex in mitochondria, leading to mitochondrial dysfunction and apoptosis [27]. (**b**) Short-term treatment with high doses of Cort enhances the formation of GR/Bag-1 complex and GR mitochondrial localization while long-term treatment with high doses of Cort reduces the mitochondrial GR and GR/Bag-1 colocalization. Chronically Cort-treated Bag-1 overexpressing mice blocked the decrease in mitochondrial GR, reversed the anhedonia-like behavior as well as the depressive-like behavior induced by chronic Cort treatment compared to wild type mice, suggesting that Bag-1 regulates resilience from depressive-like impairments [68]. GCs; Glucocorticoids, GR; Glucocorticoid Receptor, GREs; Glucocorticoid Response Elements, Bcl2; B-cell lymphoma-2, Bag-1; Bcl-2-associated athanogene, Cort; Corticosterone.

To further elucidate the underlying mechanisms, Du et al. investigated the interaction between Bcl-2 protein and GRs in response to Cort treatment and they revealed that acute treatment with low or high doses of Cort leads to increase of the GR/Bcl-2 complex into mitochondria while long-term and high-dose treatment with Cort results in downregulation of GR/Bcl-2 levels in mitochondria. Furthermore, treatment with RU486 blocked the acute and high Cort-induced effects on mitochondrial function as well as mitochondrial translocation of GRs in primary cortical neurons, suggesting that these effects are mediated through GRs [27]. The same research group carried out in vivo studies and revealed that prefrontal cortex (PFC) of treated rats with long-term high and low doses of Cort, exhibit lower mitochondrial levels of GRs while high dose Cort-treated mice exhibit also reduced mitochondrial Bcl-2 levels [27]. Based on above data, Du et al. suggested a possible role of mitochondrial GR in underlying mechanism(s) in chronic stress-induced apoptosis [64]. According to this mechanism, GR/Bcl-2 complex translocates into mitochondria and regulates mitochondrial function and neuronal viability/apoptosis in a biphasic manner; under physiological conditions, GCs enhance mitochondrial function and neuroprotection through an increase of the mitochondrial GR/Bcl-2 complex whereas under chronic stress conditions GCs attenuate mitochondrial function and induce neurotoxicity through decrease of the mitochondrial GR/Bcl2 complex. Given that Hsp70 mediates protein mitochondrial translocation [65] whereas Bag-1 attenuates nuclear translocation of GR and potentiates the anti-apoptotic function of Bcl-2 [66,67], Du et al. hypothesized that GR/Bcl2 mitochondrial translocation can be mediated via its binding to GR chaperones, heat shock protein 70/90 (Hsp70/90) and Bag-1 (Figure 2a) [64].

The role of Bag-1 in GR mitochondrial translocation was further investigated by Luo et al. who examined Cort dose and time effects on Bag-1/GR complex formation and GR mitochondrial translocation in cortical neuronal cultures and in animal studies [68]. Their data revealed that short-term treatment with high doses of Cort significantly enhanced the formation of the Bag-1/GR complex and GR mitochondrial translocation, whereas after long-term and high dose incubation of Cort, the Bag-1/GR colocalization and mitochondrial GR were reduced, implicating that Bag-1 mediates the GR mitochondrial trafficking [68]. The important role of Bag-1 in GR mitochondrial translocation was demonstrated when Bag-1 over-expressing cortical neurons blocked the Cort-induced decrease of mitochondrial GR after long-term treatment with high doses of Cort. Consistent with the in vitro results, chronically Cort- treated Bag-1 over-expressing mice blocked the decrease in mitochondrial GR, reversed the anhedonia-like behavior as well as the depressive-like behavior induced by chronic Cort treatment compared to wild type mice [68]. Taken together, GCs exhibit dose- and time-dependent effects on neuronal mitochondrial function, neuronal viability, GR mitochondrial translocation as well as on the formation of the complexes GR/Bcl-2 and GR/Bag-1 in neurons. More importantly, the above studies reveal that Bcl-2 and Bag-1 have critical roles in GR mitochondrial trafficking, and indicate that these molecules might be potential therapeutic targets for neurodegenerative diseases (Figure 2b).

### 6.3. Pheochromocytoma Cells

Li et al. investigated the role of mitochondrial GR in GC-induced apoptosis in Cort-treated PC12 pheochromocytoma cells [69]. They found that Saikosaponin D (SSD)-an oleanane-type glycoside-exerts neuroprotective effects, enhances mitochondrial function, through decreasing of intracellular Ca^+2^, closing of mitochondrial permeability transition pores (mPTPs), and restoring the depolarisation of mitochondrial membrane potentials (MMPs) while inhibits the mitochondrial apoptotic pathway. More importantly, the ability of SSD to restore the mitochondrial function and reverse the Cort-induced apoptosis was accompanied by attenuation of GR mitochondrial translocation. To further elucidate the involvement of GR-dependent pathways in the neuroprotective role of SSD, Li et al. measured the protein expression levels of the following GR-dependent mediators; GILZ (GC-induced leucine zipper), SGK-1 (glucocorticoid inducible kinase-1), NF-κB (nuclear factor kappa-light-chain-enhancer of activated B cells), IκB-α (nuclear factor of kappa light polypeptide gene enhancer in B-cells inhibitor, alpha), Akt/P-Akt (Protein kinase B/phosphorylated-Akt) and Bad/P-Bad (Bcl2 associated agonist of cell death/phosphorylated Bad) and reveal that SSD remediate the mitochondrial dysfunction through positively regulation of the major cytoprotective proteins (GILZ, SGK-1, IκΒ-α, NF-kB, P-Akt, P-Bad) [69]. Based on this data, Li et al. implicate that SSD exerts its anti-apoptotic effects through regulation of GRs mitochondrial translocation and selective activation of the GR-dependent survival pathway. To further elucidate the mechanisms involved in SSD neuroprotective effects, they also examined the role of Hsps as well as HDAC6 in Cort-induced apoptosis and they showed that Cort treatment enhanced HDAC6 expression and decreased the expression of Hsp90 while SSD treatment reversed these Cort-induced effects [69]. Given that HDAC6 enhances the affinity of the GR-GC complex via deacetylation of Hsp90 (GR chaperone) [70], the deacetylated Hsp90 may in turn accelerates its complex with Hsp70, favoring thus the translocation of cytoplasmic GR into mitochondria [71].

Subsequent work from the same group revealed that a selective HDAC6 inhibitor -HPOB (N-hydroxy-4-(2-((2-hydroxyethyl) (phenyl)amino)-2-oxoethyl) benzamide)-can also inhibit Cort- induced apoptosis and enhance mitochondrial function through blocking mitochondrial translocation of GRs in Cort-treated PC12 cells [72]. To elucidate the mechanism by which HPOB attenuates GR mitochondrial translocation, they focus on the role of HDAC6-mediated deacetylation. Their study demonstrated that HPOB triggered the hyperacetylation of Hsp70 and Hsp90 and reversed Cort-induced upregulation of HDAC6 and reduction of Hsp70 and Hsp90, implicating that HPOB-mediated acetylation of Hsps might be involved in both the trafficking and mitochondrial translocation of GR [72]. The mitochondrial membranes contain specific machineries for recognition, translocation, and membrane insertion of precursor proteins, primarily composed of TOM complex located in the outer mitochondrial membrane and TIM complex which is organized in the inner mitochondrial membrane [73]. Tom 70 receptor, component of TOM complex, preferentially recognizes preproteins with internal localization sequences, such as GR [74]. The interaction of Tom70 with multi-chaperone complexes, including Hsp90 and Hsp70 and their functional link Hsp70/Hsp90-organizing protein (Hop) allows the pre-protein import into mitochondria [75]. Proteins destined for the mitochondrial matrix are translocated through Tim22, while Mia40, alongside with the mtHsp60, selectively recognizes proteins destined for the intermembrane space [76].

**Figure 3:**
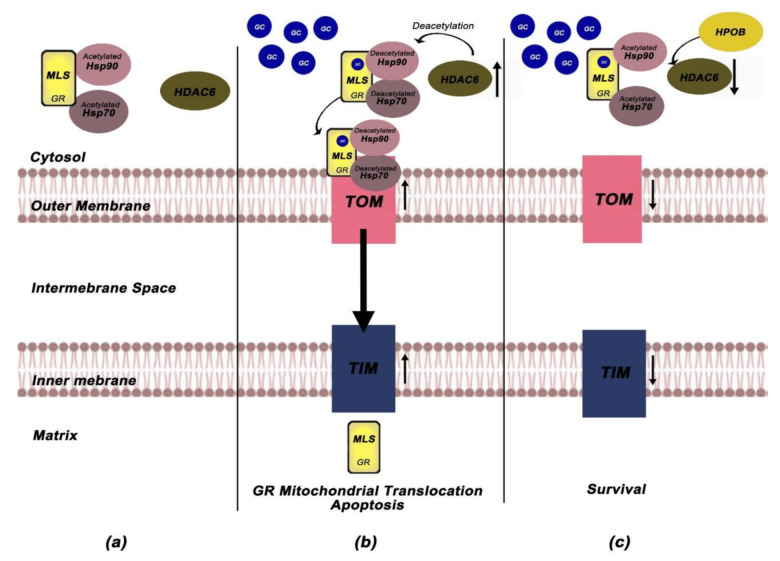
The involvement of HDAC6 and mitochondrial translocation machinery in GR import into mitochondria. (**a**) In unliganded state, GR is located into the cytoplasm complexed with low affinity to acetylated GR chaperones (Hsp70, Hsp90). (**b**) Cort treatment enhances the expression levels of HDCA6 and decreases the levels of Hsp90/70. HDCA6 triggers the deacetylation of Hsp70 and Hsp90, enhancing the affinity of GR complex for GC and favoring GR translocation into mitochondria. Cort also induces upregulation of the outer and inner membrane mitochondrial machinery (TOM and TIM), thus leading to GR import into mitochondria and inducing apoptosis processes. (**c**) HPOB treatment attenuates GR mitochondrial translocation through inhibiting HDAC6. HPOB triggers hyperacetylation of Hsp70 and Hsp90 and reverses the Cort-induced upregulation of HDCA6 and reduction of Hsp70 and Hsp90. Hsps hyperacetylation inhibits the binding between the multi-chaperone complex and Tom70, blocking the mitochondrial GR translocation and Cort-induced apoptosis [69,72]. GCs; Glucocorticoids, GR; Glucocorticoid Receptor, MLS; Mitochondrial localization signal, Hsp; Heat Shock Proteins, HDAC6; Histone Deacetylase 6.

In an in-depth study of the role of mitochondrial translocation complexes in GR mitochondrial translocation, Li et al. examined the protein levels of Tom20, Tom40, Tom70, Hop, Tim22, Mia40, and mtHsp60 in Cort-treated PC12 cells. HPOB attenuated the Cort-induced increase in Tom20, Tom40, Tim22, Mia40, and mtHsp60 expression while declined the Cort-induced binding of intracellular Tom70 and Hop to Hsp90, indicating that Hsps hyperacetylation inhibits the binding between the multi-chaperone complex and Tom70 and blocks the mitochondrial GR translocation [72]. Based on the above, Li et al. provided a mechanism according to which HPOB neuroprotective effects on GC-induced apoptosis can be mediated through inhibition of the formation of multi-chaperone translocation system that ultimately block the mitochondrial GR translocation. More importantly, their study revealed that selective HDAC6 inhibitors can be used as neuroprotective agents against GC-induced apoptosis [72]. Figure 3a–c depicts all the above experiments about the role of HDCA6 and mitochondrial translocation machinery in GR mitochondrial import while Table 2 summarizes the data regarding the association between mitochondrial GR and apoptosis-associated parameters.

Interestingly, Gallo et al. revealed that, another GR chaperone, immunophilin FKBP51 can also form complexes with GR in mitochondria of fibroblasts and rat liver [77]. FKBP51 overexpression protected cells against oxidative stress, whereas FKBP51 knockdown made them more susceptible to cell death, implicating that mitochondrial FKBP51/GR complex may prevent the deleterious effect of mitochondrial GR on cell survival due to inhibitory effect of FKBP51 on GR [77]. Taken together the above studies, it is evident that GR chaperones, including Hsp70, Hsp90, and FKBP51, and the mitochondrial translocation machinery are important regulators of GR import into mitochondria, thus influencing mitochondrial function and cell survival.

## 7. Phosphorylated Isoforms of Mitochondrial Glucocorticoid Receptor in Stress and Depression-Like Behavior

Increasing evidence indicates that stress-related neuropsychiatric disorders, may be caused by energy impairment in the brain due to mitochondrial dysfunction [78]. Adzic et al. investigated the effects of acute, chronic, and combined stress on mitochondrial GR, its phosphorylation status, *COX1* and *COX3* expression levels as well as Bcl2 family members redistribution in hippocampus (HIPPO) and prefrontal cortex (PFC) of Wistar rats [79]. Importantly, rats subjected to chronic stress exhibit GR mitochondrial accumulation in HIPPO and PFC, primarily phosphorylated at S232, in parallel with differentially regulation expression of mitochondrial *COX1* and *COX3* genes, in two tissues. HIPPO was associated with elevated levels of *COX1* and *COX3* while PFC was associated with decreased levels, indicating that the same GR phosphoisoform can regulate positively or negatively the transcriptional activity probably due to different tissue specific cofactors. Chronic stress also caused redistribution of Bcl2 family members, favoring the initiation of apoptosis signaling pathway in both tissues [79]. Their data indicate that mitochondrial GR and its phosphorylation status are tissue specific, and they are associated with alterations in *COX*s expression levels and apoptosis-related parameters. More importantly, these data make clear that GR signaling at the level of brain mitochondria is regulated by a stress-dependent manner.

Subsequent work from Adzic et al. investigated particularly the sex-specific effects of chronic stress on mitochondrial GR and its phosphorylation status in the PFC and HIPPO of rat brain [80]. Chronic stress caused GR accumulation in mitochondria of female PFC while the changes in the HIPPO were sex-specific at the levels of phosphoGRs. In this study, Adzic et al. also investigated the tissue and sex-specific effects of the antidepressant fluoxetine on the mitochondrial GR levels and its phosphorylation status [80]. Fluoxetine significantly increased mitochondrial GR in female HIPPO in parallel with increase of *COXs* expression levels probably through elevation of mitochondrial pGR232 while in males it diminished *COXs* expression and had no effect on cytochrome c oxidase activity. Their data suggest that tissue differences in brain metabolism in stress-related disorders are sex-specific and could be associated with mitochondrial GR alterations. In addition, antidepressant fluoxetine could affect mitochondrial GR and its phosphorylation status, in a region- and sex-specific manner. More importantly, mitochondrial GR signaling may be associated with gender-dependent vulnerability to stress as well as gender-specific clinical impacts of antidepressants [80]. The association between mitochondrial GR and apoptosis-related alterations in stressed animals is summarized in Table 3.

Brkic et al. investigated the role of mitochondrial GR in the PFC of male and female Wistar rats with depressive-like behavior [81]. LPS treatment initiated apoptotic cascades in both sexes differently; in females the treatment initiated both intrinsic and extrinsic apoptotic cascade, while in males only intrinsic cascade was activated. Furthermore, LPS treatment decreased levels of mitochondrial GR and increased pGR232/pGR246 ratio in males while these alterations were further associated with increase in *COX1* and *COX3* mRNA expression levels in this sex. These data, alongside with the elevated levels of Bcl2 in male PFC, imply a protective mechanism from LPS-induced apoptosis signaling pathways in this sex [81].

Subsequent similar work from Brkic et al., using HIPPO from female and male Wistar rats with depressive-like behavior, demonstrated that LPS treatment effect on mitochondrial GR was sex-specific. LPS decreased levels of mitochondrial GR, its phosphorylation at Ser232, as well as pGR232/pGR246 ratio only in females, which were further associated with decreased mRNA levels of *COX-1* and *COX-3*. This indicated an impaired oxidative metabolism in HIPPO of this sex [82]. However, the increased levels of the mitochondrial Bcl2 imply that these alterations could protect the cells from LPS-induced apoptosis [82]. The above studies suggest that mitochondrial GR alterations, *COXs* expression levels and apoptosis-related parameters are sex-specific in LPS-induced apoptosis signaling pathways in both brain tissues. Furthermore, these studies point out the important role of mitochondrial phosphorylated GR in apoptosis mediated processes in stress and depression. Table 4 summarizes the association between mitochondrial GR and apoptosis-related alterations in animals with depressive-like behavior.

## 8. Mitochondrial Glucocorticoid Receptor and Lung Inflammation

Mounting evidence supports that GCs exhibit a crucial key role in the maturation of the developing respiratory system in utero [83]. However, little is known about the involvement of mitochondrial GR in lung mitochondrial dysfunction as well as in the development of lung diseases. Recently, Simoes et al., using a mouse model of allergic airway inflammation disease, evaluated the presence of GR in mitochondria of lung epithelial cells and explore its possible role in allergic airway inflammation [84]. Their data revealed that allergic airway inflammation caused reduction in mitochondrial GRa, and OXPHOS enzyme biosynthesis including the nuclear encoded SDH (succinate-ubiquinol oxidoreductase subunit) of the OXPHOS Complex II, and the mitochondrially encoded COX-I (cytochrome oxidase subunit I) of Complex IV, especially in bronchial epithelial cells of lung rat tissues. Importantly, these alterations were associated with decrease in lung mitochondrial mass and induction of apoptosis. Further analysis in autopsies from human fatal asthma cases revealed mitochondrial GR reduction in lung epithelial cells thus enhancing the crucial role of GR in the regulation of mitochondrial function in asthma, as well as its involvement in the pathophysiology of the disease.

## 9. Mitochondrial Glucocorticoid Receptor and Hepatic Inflammation

The relationship between GCs and obesity has been extensively studied [85]. Although obesity is associated with hepatic mitochondrial dysfunction, the role of mitochondrial GR in obesity-induced mitochondrial dysfunction remains unclear. Recent evidence from Li et al., using a high-fat diet mouse model (HFD), showed that the hepatic expression of mitochondrially encoded genes, including *ATP8, COX1, CYTB, ND1*, and *ND5*, mtDNA copy number, ATP content and COX enzyme activity were lower in HFD mice compared to control mice [86]. Furthermore, both GR translocation into the mitochondria and the GR binding to the mtDNA were lower in the liver of HFD mice. In addition, PGC1a (Peroxisome proliferator-activated receptor gamma coactivator 1-alpha) mRNA and protein expression as well as GR binding to PGC1a promoter were also found to be reduced in HFD mice, suggesting that the hepatic GR may be a regulator of PGC1a expression, and both could be involved in obesity-induced mitochondrial dysfunction through regulating mtDNA expression.

In another study, Liu et al. investigated the role of mitochondrial GR in LPS-mediated inflammation in liver in parallel with the effects of a-Lipoic acid (LA) on liver metabolism [87]. LPS-treated mice exhibited decreased hepatic ATP and NADH concentrations, and mtDNA copy number, while many mitochondrially encoded genes, including *COX2, COX3, ND1, ND3, ND4, ND6, ATP6* and *ATP8*, were significantly downregulated. LPS significantly decreased GR protein expression and decreased GR binding on the mtDNA D-loop region. However, treatment with LA reversed the LPS-induced changes in liver, suggesting a liver-protective effect of LA in inflammation conditions [87]. More importantly, these data imply the involvement of mitochondrial GR in liver mitochondrial dysregulation and thus in the development of hepatic inflammation.

## 10. Mitochondrial Glucocorticoid Receptor and Thermoregulation

Evidence has shown that GR alongside with Mineralocorticoid Receptor (MR) can participate in the control of energy expenditure, through inhibition of uncoupling proteins —UCP1 and UCP3—which consist of specific mitochondrial proton transporters involved in thermoregulation and control of energy expenditure [88]. In vivo and in vitro studies by Chen et al., using thermal acclimation (TA)-exposed mice as well as TA and Cort-treated C2C12 mouse myoblast, investigated the association between GR and resistance to heat-induced hyperthermia and injury [89]. Their data revealed that heat tolerant and intolerant heat exposed mice—compared to mice not exposed to heat—exhibited reduced cytosolic levels of GR in the gastrocnemius muscles while tolerant mice exhibited elevated mitochondrial and nuclear GR. In addition, both the mitochondrial and nuclear GR levels were significantly increased in TA mice compared to control. Similar data were also obtained from TA and Cort-treated C2C12 mouse myoblasts which exhibit increased viability during heat exposure and increased mitochondrial and nuclear GR expression, further supporting the idea that GR activation in mitochondria is associated with increased resistance against heat-induced hyperthermia and injury [89]. Table 5 summarizes the association between mitochondrial GR and mitochondrial-related alterations in lung disease, hepatic inflammation, and hyperthermia.

## 11. Clinical Applications

In most disorders of the nervous system, impaired brain mitochondrial dysfunction and selective death of neuronal subtypes are detected [90]. Bcl-2 and GR chaperones, including Hsp70/90, Bag-1, and FKBP51 are known to be involved in GR mitochondrial trafficking, and therefore can modulate mitochondrial function and apoptosis-mediated processes [27,68,77]. Thus, GR chaperones in brain mitochondria have emerged as crucial therapeutic targets for the treatment of neurodegenerative diseases. Bag-1, an identified target for the actions of mood stabilizers, seems to have an important role in the development of mood disorders while therapies designed to enhance the function of Bag-1 may modulate the effects of stress hormone [68]. In addition, the selective inhibition of HDAC6 could be also a therapeutic target against GC-induced apoptosis through blocking the mitochondrial translocation of GR and the subsequent mitochondrial dysfunction and activation of intrinsic apoptosis pathway [72]. The identification of sex- specific mitochondrial metabolic enzyme regulation by the stress and by the antidepressant therapy and its differential convergence with mitochondrial GR signaling contribute to clarification of sex- dependent vulnerability to stress-related disorders and sex-specific clinical impact of antidepressants [80]. The involvement of mitochondrial GR in the pathophysiology of diseases such as lung and hepatic inflammation, reveals that mitochondrial GR could also serve as a potential therapeutic target for disease treatment [84,86,87].

## 12. Concluding Remarks

Published evidence has clearly stated that GR binds to GREs on the mitochondrial genome while GR mitochondrial translocation is associated with changes in the transcription of mitochondrially encoded OXPHOS genes, thus influencing mitochondrial function and cell viability, in parallel with its nuclear action. The effects of GCs on GR mitochondrial translocation and mitochondrial gene transcription are dose- and time-dependent. Increasing evidence supports the involvement of mitochondrial GR in apoptosis-mediated pathways and points out the role of GR chaperones (Hsp70/90, Bag-1, FKBP51) and the anti-apoptotic protein Bcl-2 in mitochondrial function and GR mitochondrial trafficking. Importantly, HDAC6-mediated deacetylation and the interaction of the Hsp70/90 with the translocation complexes of outer mitochondrial membrane (Tom) may guide GR mitochondrial translocation. The role of mitochondrial GR in mitochondrial dysfunction associated with disease development, including asthma, stress, depression, and obesity has also been determined. Interestingly, mitochondrial GR phosphorylation status has been related to stress and depression-induced apoptosis in a sex and tissue specific manner. These findings give a better understanding of the mechanisms by which mitochondrial GR can modulate mitochondrial function, cellular plasticity, and resilience. More importantly, the identification of the mechanism of GR mitochondrial trafficking could provide new molecules/targets for the treatment of neurodegenerative diseases. The involvement of mitochondrial GR in the pathophysiology of diseases, including lung and hepatic inflammation, could also provide new therapeutic opportunities.

## Figures and Tables

**Table 1:** Mitochondrial GR localization in parallel with alterations in the expression of the mitochondrially encoded genes.

Cell Culture/Animal Tissue	Treatment	Mitochondrial GR ^1^	Mitochondrially Encoded Genes	Refs
Rat C6 glioma cells	Dex ^2^	mt ^4^ GR↓	COX-1↑	[49]
	Dex + RU486	mt GR↑	COX-1↓	
HepG2 hepatocarcinoma cells	Dex	mt GR↑	12S rRNA, 16S rRNA, ND1, ND2, ND3, ND4, CYTB, ATP6, ATP8, COXI↑	[51]
Rat hippocampus	Acute stress	mt GR↑	ND1, ND3, ND6, ATP6↓	[52]
	Chronic stress	mt GR↑	ND6↑	
	Low dose Cort ^3^	GR binding to D-loop↑	ND3, ND4, COX2, ND4L, ATP6, ATP8, ND5, COX3, COX1, CYTB↑ ND1, ND2↓	
	High dose Cort	GR binding to D-loop↓		

^1^ GR; Glucocorticoid Receptor, ^2^ Dex; Dexamethasone, ^3^ Cort; Corticosterone, ^4^ mt; mitochondrial.

**Table 2:** Mitochondrial GR and apoptosis-associated parameters.

Cell Culture/Animal Tissue	Treatment	Mitochondrial GR ^1^	Mitochondrial Parameters/Apoptosis	Refs
T-lymphoid GC-sensitive cell lines	Dex ^2^	mt ^4^ GR↑	apoptosis↑	[60]
T-lymphoid GC-resistance cell lines			apoptosis↓	
CD4 + CD8+ double-positive GC-sensitive thymocytes	Short-term/High dose Dex	mt GR↑	mt membrane potential↓ apoptosis↑	[61]
Mouse thymocytes	Short-term/High dose Dex	mt GR↑ mt GR/Bak, GR/Bcl-xL↓ mt GR/Bim↑	mt Bax↑ cleaved caspase- 3, 9, 8↑ cytochrome c↑ apoptosis↑	[62]
Bovine blood neutrophils	Dex		A1 expression↑ Bak expression↓ caspase 9 activity↓ membrane stability↑ apoptosis↓	[63]
	Dex + RU486		A1 gene expression↓ Bak gene expression↑ caspase 9 activity↑	
Primary cortical neurons	Short-term/Low and High Dose Cort ^3^	mt GR↑mt GR/Bcl2↑	mt oxidation↑ mt membrane potential↑ mt calcium holding capacity↑ mt Bcl2↑ apoptosis↓	[27]
	Long-term/High Dose Cort	mt GR↓ mt GR/Bcl2↓	mt oxidation↓ mt membrane potential↓ mt calcium holding capacity↓ mt Bcl2↓ apoptosis↑	
Mouse prefrontal cortex	Long-term/Low Dose Cort	mt GR↓		
	Long-Term/High Dose Cort	mt GR↓	mt Bcl2↓	
Primary cortical neurons	Short-term/High Dose Cort	mt GR↑ mt GR/Bag-1↑ t ^5^ GR↓	tBag-1↓	[68]
	Long-Term/High Dose Cort	mt GR↓ mt GR/Bag-1↓ tGR↓	tBag-1↓	
Mouse prefrontal cortex	Long-term/High Dose Cort	mt GR↓ tGR↓		
PC12 pheochromocytoma cells	Cort	mt GR↑	apoptosis↑ intracellular Ca^2+^↑ DNA fragmentation↑ mt permeability transition pores↑ mt depolarizationcaspase- 3, 9↑ cytochrome c↑ GILZ, SGK-1, IκΒ-α, HDAC6↑ NF-κΒ, P-Akt, P-Bad, Hsp90↓	[69]
PC12 pheochromocytoma cells	Cort	mt GR↑	caspase-3 activity↑ LDH ^6^ leakage↑ mt permeability transition pores↑ mt depolarizationapoptosis↑ cleaved caspase 3, cytochrome c↑ mt Fis1, DRP1, Bax, HDAC6↑ Hsp90, Hsp70↓ Tom20, Tom40, Tom70, Hop↑ Tom70 and Hop/Hsp90↑ Tim22, Mia40, mtHsp60↑	[72]

^1^ GR; Glucocorticoid Receptor, ^2^ Dex; Dexamethasone, ^3^ Cort; Corticosterone, ^4^ mt; mitochondrial, ^5^ t; total^, 6^ LDH; lactate dehydrogenase.

**Table 3:** Mitochondrial GR (total GR and phosphoGR isoforms) and apoptosis-related alterations in stressed animals.

Animal Models	Treatment	Sex	Tissue	Mitochondrial GR ^1^ (Total and Phospho)	Mitochondrial Gene Expression/Activity	Apoptosis Parameters	Refs
Stressed Wistar rats	Acute Stress (Acute immobilization for 30 min)	Male	HIPPO ^2^	mt ^7^ GR levels↓ mt p ^9^ GRT171↑ mt pGRS232↓ mt pGRS246↓		mt Bax↓	[79]
			PFC ^3^	mt pGRS232↑ mt pGRT171↓		mt Bcl2↓ cyt ^8^ Bcl2↑ cyt Bax↑	
	Chronic Stress (Chronic isolation stress for 21 days)		HIPPO	mt GR levels↑ mt pGRS232↑ mt pGRT171↓	mt COX1, COX3↓	mt Bcl2↓ cyt Bcl2↑ mt Bax↓ DNA fragm ^10^↑	
			PFC	mt GR levels↑ mt pGRS232↑	mt COX1, COX3↑	mt Bcl2↓ cyt Bcl2↑ DNA fragm↑	
	Combined Stress (Chronic isolation for 21 days followed by 30 min immobilization		HIPPO	mt GR levels↑ mt pGRS232↑ my pGRS246↓	mt COX1, COX3↓	mt Bcl2↓ cyt Bcl2↑ mt Bax↑ DNA fragm↑	
			PFC	mt GR levels↑ mt pGRS232↑ mt pGRS246↑	mt COX1, COX3↑	mt Bcl2↓ cyt Bcl2↑ mt Bax↑ DNA fragm↑	
Stressed Wistar rats	Chronic Stress (Chronic isolation stress for 21 days)	Male	HIPPOw/o ^4^ FLUO ^6^	mt pGR171/GR↓ mt pGR246/GR↓	cytochrome c oxidase↓		[80]
			HIPPOw ^5^ FLUO	mt GR↑ mt pGR171/GR↑ mt pGR232/GR↑ mt pGR246/GR↑	mt COX1, COX3↓		
			PFC w/o FLUO	mt pGR171/GR↓	cytochrome c oxidase↓		
			PFC w FLUO	mt GR↑ mt pGR246/GR ratio↓			
		Female	HIPPO w/o FLUO	mt pGR246/GR ratio↑			
			HIPPO w FLUO	mt pGR232/GR↑	mt COX1, COX 3↑ cytochrome c oxidase↑		
			PFC w/o FLUO	mt GR↑ mt pGR171/GR↓	mt COX1, COX3↑ cytochrome c oxidase↑		
			PFC w FLUO	mt GR↓ mt pGR171/GR↑ mt pGR232/GR↑ mt pGR246/GR↑			

^1^ GR; Glucocorticoid Receptor, ^2^ HIPPO; Hippocampus, ^3^ PFC; Prefrontal Cortex, ^4^ w/o; without, ^5^ w; with, ^6^ FLUO; Fluoxetine, ^7^ mt; mitochondrial. ^8^ cyt; cytoplasmic, ^9^ p; phoshoisoform, ^10^ fragm; fragmentation.

**Table 4:** Mitochondrial GR (total GR and phosphoGR isoforms) and apoptosis-related alterations in animals with depressive-like behavior.

Animal Models	Treatment	Sex	Tissue	Mitochondrial GR ^1^ (Total and Phopho ^4^)	Mitochondrial Gene Expression	Apoptosis Parameters	Refs
Wistar rats with depressive-like behavior	7-day LPS ^7^ treatment	Male	PFC ^2^	mt ^5^ GR↓ mt pGR232/pGR246↑ mt pGR246↓	mt COX1, COX3↑	cyt ^6^ cleaved PARP-1↑ cyt cleaved caspase 3↑ mt Bcl2↑ cyt Bcl2↓ mt Bax↓ cyt Bax↓ mt truncated BID↓ cyt truncated BID↑	[81]
		Female		mt GR↓		cyt cleaved PARP-1↑ mt cleaved caspase-3, 9↓ cyt cleaved caspase-8↑ mt full length BID↑ mt truncated BID↑ cyt full length BID↑ cyt truncated BID↑ cyt Bcl2↓cyt Bax↓	
Wistar rats with depressive-like behavior	7-day LPS treatment	Male	HIPPO ^3^	mt pGR232↑		cyt cleaved PARP-1↓ cyt cleaved caspase 3↓ mt Bcl2↓ cyt Bcl2↓	[82]
		Female		mt GR↓ mt pGR232↓ pGR232/pGR246↓	mt COX1, COX3↓	mt Bcl2↑ cyt Bax↓ cyt Bcl2↓	

^1^. GR; Glucocorticoid Receptor, ^2^ PFC; Prefrontal Cortex, ^3^ HIPPO; Hippocampus, ^4^ p; phosphoisoform, ^5^ mt; mitochondrial, ^6^ cyt; cytoplasmic, ^7^ LPS; lipopolysaccharide.

**Table 5:** Mitochondrial GR and mitochondrial-related alterations in lung disease, hepatic inflammation, and hyperthermia.

In Vivo/In Vitro System	Treatment	Sex	Tissue	Mitochondrial GR ^1^	Mitochondrial Gene Expression Levels/Enzyme Activity	Mitochondrial-Related Alterations	Refs
Allergic airway inflammation mouse model	Ovalbumin	Female	Lung tissues	mt GR↓	mt ^4^ COX1↓ citrate synthase↓	SDH↓ mt ERβ↓ cytochrome c↑ cyt un-cleaved caspase-9↓ cleaved caspase-3↑ PARP↓ mt mass↓	[84]
Human subjects with asthma		Male and Female		mt GR↓		mt ERβ↓	
High-fat diet mouse model	7 weeks High-fat Diet	Male	Liver tissues	mt GR↓	mt ATP8, COX1, CYTB, ND1, ND5↓ COX↓	ATP content↓ mtDNA copy number↓ PGC1a↓ GR binding to the D-loop↓ GR binding to the PGC1a↓	[86]
Inflammation mouse model	5 days LPS^2^ treatment	Male			mt ATP8, COX1, CYTB, ND1, ND5↓ Complex IV, V↓	ATP, NADH↓ mtDNA copy number↓ Sirt3↓ GR binding to the D-loop↓	[87]
Heat tolerant mice	Heat Exposure	Male	Muscle tissues	p ^5^ GRS211↑ mt GR↑			[89]
Thermal acclimation (TA)-exposed mice				pGRS211↑mt GR↑			
C2C12 mouse myoblast	Cort^3^ heat exposure		Myoblasts	mt GR↑			

^1^ GR; Glucocorticoid Receptor, ^2^ LPS; Lipopolysaccharide, ^3^ Cort; Corticosterone, ^4^ mt; mitochondrial, ^5^ p; phosphoisoform.

## Data Availability

Not applicable.
